# Probiotics and Coronavirus disease 2019: think about the link

**DOI:** 10.1017/S000711452000361X

**Published:** 2021-11-28

**Authors:** Suresh Kumar Angurana, Arun Bansal

**Affiliations:** Division of Pediatric Critical Care, Department of Pediatrics, Advanced Pediatrics Centre, Postgraduate Institute of Medical Education and Research (PGIMER), Chandigarh 160012, India

**Keywords:** Coronavirus disease 2019, Probiotics, Gut microbiome, Dysbiosis, Cytokine storm

## Abstract

The pandemic of Coronavirus disease 2019 (COVID-19) is rapidly progressing, causing significant morbidity and mortality. Various antiviral drugs, anti-inflammatory drugs and immunomodulators have been tried without substantial clinical benefits. The severe and critical cases of COVID-19 disease are characterised by gut microbiome dysbiosis, immune dysregulation, hyper-inflammation and hypercytokinaemia (cytokine storm). Therefore, the strategies which target these pathophysiological processes may be beneficial. Probiotics are one such strategy that exerts beneficial effects by manipulation of the gut microbiota, suppression of opportunistic pathogens in the gut, decreasing translocation of opportunistic organisms, activation of mucosal immunity and modulation of the innate and adaptive immune response. Probiotics are the potential candidates to be tested in moderate and severe cases of COVID-19 due to several beneficial effects, including easy availability, easy to administer, safe and economical to use.

## Graphical abstract

**Figure:**
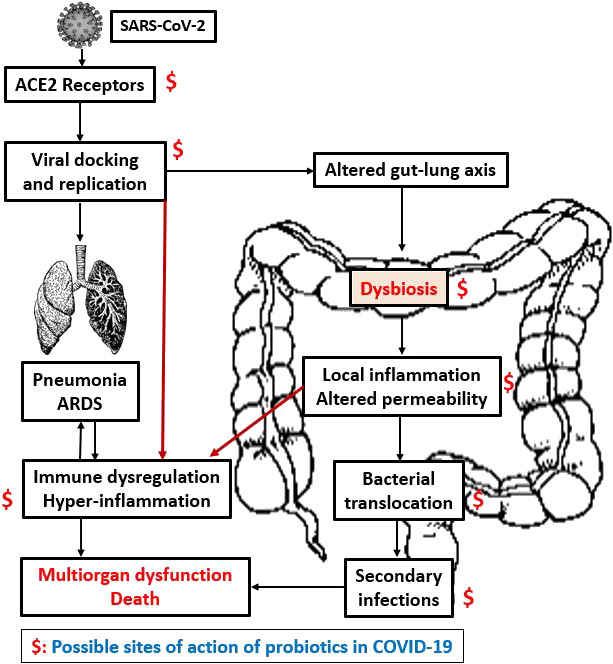

The pandemic of severe acute respiratory syndrome Coronavirus-2, causing Coronavirus disease 2019 (COVID-19), is rapidly progressing with 25 118 689 cases and 844 312 deaths all over the world with a mortality rate of 3·4 % (the WHO dashboard as on 31 August 2020). The main reasons for the massive impact of COVID-19 are lack of preparedness for unprecedented and unexpected spread, intrinsic virulence of the pathogen and its contagiousness, asymptomatic spreaders, lack of effective vaccine, and lack of proven and effective antiviral drugs^(1)^.

## Hyper-inflammation in Coronavirus disease 2019

The COVID-19 has a heterogeneous presentation and clinical course. The majority of cases are either asymptomatic or have mild symptoms^(2–5)^. The severe or critical diseases have been documented in 2–5 % of cases. The mortality among patients admitted to intensive care units ranges from 30 % to more than 60 %^(6–9)^. The severe COVID-19 is characterised by immune dysregulation, hyper-inflammation and hypercytokinaemia (cytokine storm or cytokine release syndrome). The level of cytokines (IL-1*β*, IL-1ra, IL-2, IL-6, IL-10, IL-17, TNF, interferon-*γ*, granulocyte monocyte-colony stimulating factor, interferon-induced protein-10 and monocyte chemotactic protein-3) is higher in non-survivors or severe COVID-19^(10–16)^. Therefore, with the onset of a cytokine storm, the antiviral drugs alone may not be sufficient and it is suggested to add anti-inflammatory treatment^(1,16,17)^.

In late April 2020, clinicians from the United Kingdom (UK) reported eight previously healthy children who presented with cardiovascular shock, fever and hyper-inflammation^(18)^. Subsequently, Pediatric inflammatory multisystem syndrome temporally associated with SARS-CoV-2 (PIMS-TS), multisystem inflammatory syndrome in children (MIS-C), Kawasaki disease and Kawasaki-like syndrome have been reported from the countries with high load of COVID-19 like UK, France, Italy and the USA^(14,15,18–25)^. Most of these children were treated with intravenous immunoglobulin (IVIG) and/or steroids.

## Gut–lung axis and gut microbiome in Coronavirus disease 2019

In addition to alveolar epithelial cells, enterocytes also express angiotensin-converting enzyme 2 receptors and SARS-CoV-2 RNA has been demonstrated in the faeces of infected patients^(16,26–28)^. Also, SARS-CoV-2 can invade enterocytes, and therefore, the gut may act as a reservoir for the virus^(29,30)^. The interaction of SARS-CoV-2 with angiotensin-converting enzyme 2 receptors in the gut may be responsible for the gastrointestinal symptoms, which are noted in 12–60 % cases of COVID-19, and is associated with increased disease severity^(31)^.

There is evidence available to indicate the crosstalk between gut microbiota and lung (gut–lung axis). This axis maintains the host homoeostasis and may be altered in diseased states. The gut–lung interaction is bidirectional, and the gut plays a vital role in influencing lung disease. In diseased states, the gut microbiome is disturbed (dysbiosis), leading to translocation of endotoxins and microbial metabolites from the gut, which can then impact the lung through the bloodstream. Similarly, when inflammation occurs in the lungs (e.g. respiratory viral infections), it causes perturbations in the gut microbiome (gut–lung axis)^(16,32,33)^.

This bidirectional gut–lung interaction may be playing an important role in COVID-19, leading to increased disease severity. In extreme of ages and among those with underlying co-morbid conditions, the gut microbiota is less in diversity leading to gut dysbiosis^(34–37)^. This could be one of the reasons for the increased disease severity of COVID-19 among cases with the extreme of ages and in those with underlying co-morbid medical conditions^(38,39)^. It has been demonstrated that the gut microbiome is altered in cases with COVID-19 in terms of a significant reduction in diversity and number of beneficial gut microbiota and an increase in the number of opportunistic pathogens^(16,40,41)^. This can ultimately lead to gut dysbiosis, translocation of pathogenic organisms across the gut mucosa, secondary bacterial infections, enhanced inflammatory response, multiple organ dysfunction and poor clinical outcome. Zuo *et al*.^(41)^ performed metagenomic sequencing analyses on the faecal samples of COVID-19 patients (*n* 15) from the time of admission until discharge and compared them with fifteen healthy controls. The authors demonstrated that these patients had significant depletion of beneficial commensals and enrichment of opportunistic pathogens at the time of admission as well as at all time points during the hospital stay when compared with controls. Similarly, Gu *et al*.^(40)^, in a cross-sectional study involving COVID-19 patients (*n* 30), demonstrated that the gut microbiome (faecal sample collected at admission) of COVID-19 cases had significantly reduced bacterial diversity, a lower count of beneficial symbionts and a higher number of opportunistic pathogens (*Streptococcus*, *Rothia*, *Veillonella* and *Actinomyces*).

Therefore, the heightened or dysregulated inflammation and gut dysbiosis may be the major pathophysiological processes in cases with COVID-19 leading to increased severity of illness and poor clinical outcomes.

## Current treatment strategies for Coronavirus disease 2019

**Fig. 1.**
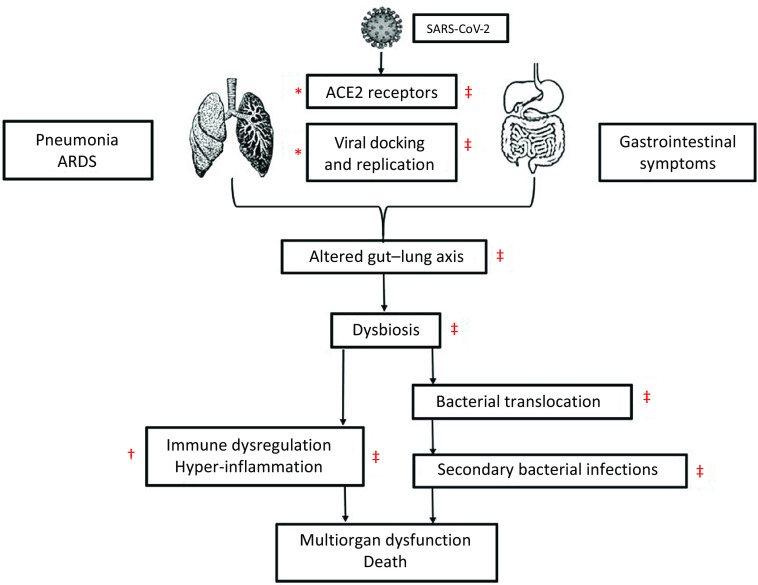
The figure shows a cascade of the pathogenesis of COVID-19 and site of action of antiviral, anti-inflammatory drugs and probiotics. The antiviral drugs act at viral docking, entry and viral replication stage (*). Anti-inflammatory drugs act at the stage of hyper-inflammation (†). Probiotics may act at multiple stages as viral docking, entry and replication, restoration of gut microbiota and gut–lung axis, reduction in bacterial translocation and secondary bacterial infection, and modulation of hyper-inflammation (cytokine storm) (‡).SARS nCoV-2, severe acute respiratory syndrome Coronavirus-2; ACE2, angiotensin-converting enzyme 2;

The therapies such as antiviral drugs that inhibit the viral entry and/or replication (remdesivir, favipiravir and lopinavir–ritonavir) and drugs/strategies that modulate the cytokine storm (hydroxychloroquine, chloroquine, azithromycin, JAK inhibitors, IL-6 inhibitors, IL-1 inhibitors, anti-TNF-*α* agents, corticosteroids, IVIG, tocilizumab, colchicine and plasma therapy) have been the focus of research in COVID-19 but without much clinical success^(42–51)^ (Fig. 1).

## Probiotics in Coronavirus disease 2019

In the absence of proven effective therapy for COVID-19, the treatment is mainly supportive. Therefore, the alternative strategies that target pathophysiological pathways in patients with COVID-19 need to be tested. One such approach is probiotics. The WHO and FAO defined probiotics as ‘live microorganisms which, when administered in adequate amounts, confer a health benefit on the host.’ Many different micro-organisms have been considered as probiotics, including *Lactobacillus* species (*L. acidophilus, L. casei* and *L. rhamnosus*); *Bifidobacterium* species (*B. bifidum, B. longum* and *B. lactis*); *Enterococcus* species (*E. faecalis* and *E. faecium*) and *Saccharomyces* (*S. boulardii* and *S. cerevisiae*). The most commonly used strains are *Lactobacillus* and *Bifidobacterium* ^(52)^.

### Restoration of the gut microbiome by probiotics

Probiotics exert their beneficial effects through various mechanisms including manipulation and restoration of gut microbiota, enhancement of intestinal barrier function, competition with pathogens for adhesion to gut epithelium and nutrition, suppression of opportunistic pathogens, production of antimicrobial substances, decrease in translocation of opportunistic organisms, activation of mucosal immunity and modulation of the innate and adaptive immune response^(29,52–57)^. These actions of probiotics have been proven in various experimental^(58–60)^ and clinical studies^(55,61–66)^.

It is important to note that not all probiotic strains exert the same actions^(29)^. The combination of multiple strains may exert one or a few of these actions and thus act synergistically. Therefore, multistrain probiotics could be more effective than mono-strain probiotics^(67)^. A review comparing single strain and multistrain probiotics demonstrated that the multistrain probiotics had greater efficacy than individual strains, including single strains that were components of the mixtures themselves^(68)^.

Due to the beneficial effects of probiotics on gut microbiota, they have been tested to prevent various infective complications among hospitalised patients. Many strain of probiotics has been demonstrated to be safe and effective in the prevention of Clostridium difficile-associated diarrhoea among hospitalised children and adults^(69,70)^ and secondary bacterial infections including ventilator-associated pneumonia in critically ill patients^(71–74)^, postoperative infections (respiratory, urinary tract and wound infections) in patients undergoing gastrointestinal surgery^(75)^ and candida colonisation in the gastrointestinal tract and invasive candidiasis in critically ill children^(52–54)^. Systematic reviews and meta-analysis involving critically ill adults demonstrated that probiotic supplementation has been associated with decreased risk of nosocomial infections, lesser gastrointestinal complications, lower mortality and shortened ICU stays^(76–78)^. Among preterm neonates, a meta-analysis (*n* 8000) demonstrated that supplementation with certain probiotics resulted in a reduction in necrotising enterocolitis, nosocomial sepsis and all-cause mortality^(79)^.

Since the gut microbiome is altered in cases with COVID-19^(16,40,41)^, some strain of probiotics may help in restoring the gut microbiota, maintain a healthy gut–lung axis, lesser translocation of pathogenic bacteria across gut mucosa and lesser chances of secondary bacterial infections. Similar actions have been demonstrated in several experimental and clinical studies^(55,58–66,80,81)^. The findings of these studies can be extrapolated and need to be tested in cases with COVID-19.

### Modulation of inflammation by probiotics

The role of probiotics in the modulation of inflammation has been demonstrated in clinical studies involving children and adults in health and several diseased states^(55,62,63,65,66,82–85)^. Kazemi *et al*.^(82)^, in a recent systematic review and meta-analysis, analysed 167 clinical trials that investigated the effect of oral probiotics or synbiotic (for >1 week) on inflammatory markers (C-reactive protein, IL-1*β*, IL-4, IL-6, IL-8, IL-10, IL-12, TNF-*α*, interferon-*γ* and transforming growth factor-*β*) among healthy individuals and in various disease conditions. Authors noted that some probiotics lead to decrease in C-reactive protein levels in healthy individuals, metabolic disorders, inflammatory bowel disease, arthritis and critically ill condition; decrease in TNF-*α* levels in healthy individuals, fatty liver, inflammatory bowel disease and hepatic cirrhosis; increase in IL-6 in cirrhosis and renal failure; and increase in IL-10 in arthritis. In another meta-analysis (eight clinical trials, 1337 patients with diabetes), it has been demonstrated that probiotics and synbiotic supplementation significantly decreased C-reactive protein and TNF-*α* levels but did not affect IL-6 levels^(83)^. These meta-analyses suggest that supplementation with probiotics leads to a decrease in pro-inflammatory markers (hs-C-reactive protein, TNF-a, IL-6, IL-12, IL-4 and TNF-*α*) and an increase in anti-inflammatory cytokines (IL-10) in healthy and several diseased states^(82–84)^.

In an RCT (*n* 100), Angurana *et al*.^(55)^ demonstrated that administration of a multistrain probiotic (*Lactobacillus paracasei, L. plantarum, L. acidophilus, L. delbrueckii, Bifidobacterium longum, B. infantis, B. breve* and *Streptococcus salivarius*) to critically ill children with severe sepsis for 7 days resulted in a significant decrease in levels of pro-inflammatory cytokines (IL-6, IL-12p70, IL-17 and TNF-*α*) and increase in anti-inflammatory cytokines (IL-10 and transforming growth factor-*β* 1) as compared with placebo. Other studies also demonstrated that certain probiotics were useful in the modulation of inflammation in critically ill patients^(66,85)^.

As cytokine storm occurs in patients with severe COVID-19, the effect of probiotics on pro-inflammatory and immunoregulatory cytokines allows viral clearance and minimise immune response-mediated tissue damage in the lungs and other organs. So, these immune-modulatory effects of probiotics might be relevant to prevent lung injury, acute respiratory distress syndrome (ARDS) and multiple organ dysfunction syndrome which are significant complications of COVID-19^(29)^.

### Antiviral effects of probiotics

The antiviral effects of probiotics have been demonstrated in experimental and clinical studies. Few experimental studies showed that various strains of probiotics exert antiviral effect by impairing virus entry into human cells and preventing viral replication^(86–89)^. Ang *et al*.^(86)^, in an *in vitro* study involving human skeletal muscle and colon cell lines, demonstrated that *Lactobacillus reuteri Protectis* showed significant dose-dependent antiviral activity against Coxsackievirus type A strains 6 and 16 and enterovirus 71. Lee *et al*.^(87)^ studied the *in vitro* (Vero cell) antiviral activity of probiotics against Rotavirus and demonstrated that *B. longum* and *L. acidophilus* have an inhibitory effect on the Rotavirus. Similarly, other studies also showed the antiviral and immunogenic action of probiotics (*Lactobacillus* and *Bifidobacterium*) against the Influenza virus, Respiratory syncytial virus and Rotavirus^(90–94)^.

Viruses are the most frequent cause of upper respiratory tract infections. A Cochrane review (twelve RCT with 3720 participants, including children and adults) showed that probiotics were superior to placebo in decreasing the number of participants experiencing episodes of acute upper respiratory tract infections, mean duration of an episode of acute upper respiratory tract infections, antibiotic use and cold-related school absenteeism^(95)^. In influenza viral infection, probiotics have been shown to modulate respiratory immunity and had a beneficial impact on virus clearance and inflammatory-mediated lung injury^(96)^.

Recently, Anwar *et al*.^(88)^, in a computational docking study, demonstrated that three metabolites of *Lactobacillus plantarum* (Plantaricin W, Plantaricin JLA-9 and Plantaricin D) significantly blocked the binding of SARS-CoV-2 with angiotensin-converting enzyme 2 receptors (blocking the viral entry into the cells) and suggested antiviral property of *Lactobacillus plantarum* against SARS-CoV-2.

Therefore, probiotics may act as antiviral agents by interfering with the viral entry into cells and/or inhibit virus replication. This may lead to a reduction in the dissemination of SARS-CoV-2 in the respiratory tract and gut. Also, with the restoration of the gut and respiratory microbiota, immune function and gut–lung axis, the course of COVID-19 may be altered^(29)^.

## Safety of probiotics

Most of the probiotic strains are generally safe even in the vulnerable population and in an intensive care setting, including neonates, children and adults. Many probiotic strains have shown beneficial effects in these settings^(52–55,61–66,69–79,82–85,97)^. However, there are few case reports of probiotic-associated bacteraemia, infective endocarditis, liver abscesses and fungaemia. These are majorly reported in severely debilitated cases, premature babies and immunocompromised patients who were treated with preparations lacking adequate quality control^(98–104)^.

It has been suggested to exercise caution while using probiotics in the presence of a one major risk factor (immunocompromised state and premature infants) or more than one minor risk factor (central venous catheter, cardiac valvular disease, impaired intestinal epithelial barrier, administration of probiotics by jejunostomy and probiotics with properties of high mucosal adhesion or known pathogenicity) because of the risk of probiotics-sepsis^(80,105)^.

Other safety concerns are the genetic transfer of antimicrobial resistance from probiotic strains to pathogenic organisms in the gut (particularly *Staphylococcus aureus* and *Enterococcus*)^(106,107)^, deleterious metabolic activities and excessive immune stimulation in susceptible individuals^(52,80)^. The use of probiotics may be limited in the presence of immunocompromised states or premature neonates because of safety concerns, as discussed above. The supplementation with probiotics may not be possible in whom enteral administration is contraindicated.

## The rationale for using probiotics in Coronavirus disease 2019

Several clinical trials of antiviral and anti-inflammatory drugs are in progress to evaluate the therapeutic effects of these drugs in patients with COVID-19^(108)^. The site of action of currently used antiviral and anti-inflammatory medications is at limited steps in the pathogenesis of COVID-19 (Fig. 1). The probiotics, such as *Lactobacillus* and *Bifidobacterium*, possibly act at multiple levels in this cascade of COVID-19. They act through antiviral actions^(86–96)^, restoration of the gut microbiome^(55,58–66,80,81)^, modulation of inflammation/cytokine storm (anti-inflammatory)^(55,62,63,65,66,82–85)^ and prevention of secondary bacterial and fungal infections^(52–54,69–75)^ (Fig. 1). Though the evidence for the rationale of using probiotics to treat COVID-19 comes from indirect evidence, these actions of probiotics may help in the prevention of and/or alleviation of COVID-19 related symptoms and complications. Moreover, probiotics are readily available, easy to administer (oral administration), relatively safe and economical compared with antiviral drugs, immunomodulators or other strategies tested in COVID-19^(1)^.

Therefore, probiotics, due to their mechanisms of action, are a potential strategy that need to be tested in moderate to severe cases with COVID-19 by conducting well-designed clinical trials. Few registered trials are going on to evaluate the possible beneficial effects of probiotics supplementation in cases with COVID-19 (NCT04366180, NCT04368351 and NCT04366089).

A better understanding of the pathophysiology of COVID-19 and the results of the clinical trials will guide further on the efficacy, benefits and safety of probiotics in COVID-19. Despite these limitations, considering various mechanisms of actions of probiotics on the regulation of gut dysbiosis and anti-inflammatory activity, and clinical evidence about the beneficial effects of probiotics in critically ill adults and children, probiotics are a potential therapeutic strategy that need to be tested in moderate to severe cases with COVID-19.

### Conclusion

Some probiotics may have a beneficial role in the treatment of COVID-19 patients due to their antiviral activity, ability to restore gut microbiome, modulate inflammation, ready availability, ease of administration, relatively safe and economical. The recommendations regarding the strain, dose and duration of probiotic are lacking. However, given the large experience of clinical usage, and evidence for beneficial effects in the various clinical settings, *Lactobacillus* and *Bifidobacterium* can safely be used.
